# Inverted U-shaped relationship between diverticular width and gastrointestinal bleeding in symptomatic Meckel's diverticulum children: a retrospective single-center study

**DOI:** 10.3389/fped.2026.1774572

**Published:** 2026-05-15

**Authors:** Xufeng Gao, Qiangqiang Cui, Wei Liu, Hongwei Xi

**Affiliations:** Department of General Surgery, Children’s Hospital of Shanxi, Taiyuan, Shanxi, China

**Keywords:** child, diverticular width, gastrointestinal bleeding, heterotopic mucosa, Meckel's diverticulum

## Abstract

**Objective:**

Meckel's diverticulum (MD) is the most common congenital gastrointestinal malformation in children, with gastrointestinal bleeding being the most frequent associated complication. While existing studies have primarily focused on ectopic mucosa and diverticulum length as predictors of complications, no research has specifically evaluated the association between diverticulum width and MD symptoms, particularly gastrointestinal bleeding. This study aimed to determine whether diverticulum width is an independent predictor of gastrointestinal bleeding risk in symptomatic patients, and to evaluate its potential utility for individualized surgical decision-making.

**Methods:**

This retrospective cohort study investigated the association between diverticulum width and the risk of gastrointestinal bleeding in children with symptomatic Meckel's diverticulum. To do this, we conducted an in-depth analysis of the relationship between diverticulum width and gastrointestinal bleeding risk using multivariate logistic regression models, generalised additive models and two-stage linear regression models.

**Results:**

A total of 310 paediatric patients with symptomatic Meckel's diverticulum were ultimately enrolled based on inclusion and exclusion criteria. After adjusting for potential confounding factors, diverticular width was found to be an independent predictor of gastrointestinal bleeding risk. A inverted U-shaped relationship was observed between diverticular width and gastrointestinal bleeding risk, with the highest risk occurring at approximately 1.25 cm. Furthermore, a significant interaction was identified between ectopic mucosa and diverticular width in modulating gastrointestinal bleeding risk.

**Conclusion:**

In paediatric patients with symptomatic Meckel's diverticulum, the width of diverticula shows an inverse U-shaped relationship with the risk of gastrointestinal bleeding, with the highest risk occurring at around 1.25 cm. The presence of ectopic mucosa modulates this relationship through an interactive effect. These findings could help to improve risk stratification and inform surgical decision-making in cases of Meckel's diverticulum.

## Introduction

Meckel's diverticulum (MD) is the most prevalent congenital anomaly of the gastrointestinal tract in children, resulting from incomplete regression of the vitelline (omphalomesenteric) duct during embryonic development. A true diverticulum, MD is estimated to occur in approximately 2% of the population (1–7). Although many individuals with MD remain asymptomatic, an appreciable minority develop clinical complications: roughly 15% of affected persons become symptomatic, and children under 3 years of age account for approximately 50% of symptomatic cases (3, 5). Gastrointestinal bleeding is the most frequent presenting complication of MD; in a multicenter series of 151 children, bleeding comprised 37.8% of symptomatic presentations, underscoring its clinical importance (1, 5). Previous studies have emphasized the role of heterotopic mucosa and diverticular length as predictors of complications (2–6). Emerging evidence also suggests that other morphological features of MD may influence clinical outcomes (7–9). For example, Hernández et al. (5) reported that symptomatic diverticula tended to have greater length and base width compared with incidentally discovered lesions. However, the specific and independent contribution of diverticular width to bleeding risk has not been comprehensively examined. Accordingly, this retrospective single-center study was designed to determine whether diverticular width is independently associated with gastrointestinal bleeding risk in symptomatic patients with MD, and characterize the form of that association (linear vs. non-linear).

## Material and methods

### Study design and participants

**Figure 1:**
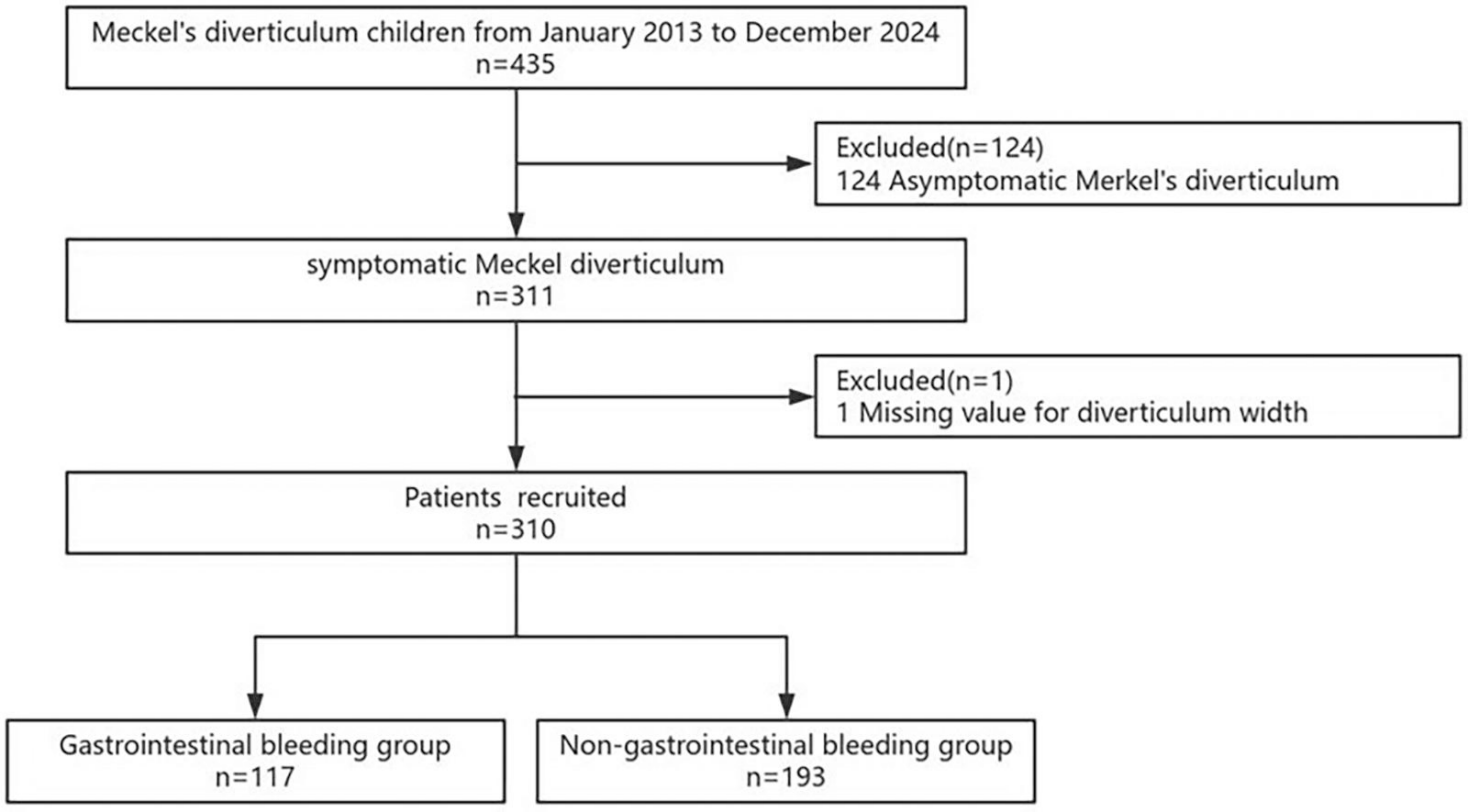
Illustrates the patient selection flowchart.

We performed a retrospective review of children with MD who underwent surgery at our tertiary hospital between 1 January 2013 and 31 December 2023. Eligible patients were identified through the hospital electronic medical records; Patients with missing value for diverticulum width and asymptomatic MD were excluded. Data extraction for research purposes was performed on 1 August 2025. All patients in this study underwent surgical treatment. Figure 1 illustrates the patient selection flowchart.

### Covariates

Various potential covariates were assessed, including sex, age, symptoms (e.g., bloody stools, vomiting, abdominal bloating and fever), Meckel' diverticular height (cm), Diverticular Width (cm), the presence or absence of histopathology, mesodiverticular band, distance from the ileocecal junction, gastrointestinal bleeding, and laboratory examination results [haemoglobin (normal range 110–140 g/L), white blood cell count (WBC), neutrophil percentages, absolute neutrophil count, red blood cell count (RBC), platelet count, C-reactive protein, and albumin (normal range 40–54 g/L)]. The width and height of Meckel's diverticulum were measured using a ruler and recorded in surgical reports or gross pathological descriptions.

### Ethics statement

During and after the data collection phase, the authors were unable to access personally identifiable participant information. All data handling and storage complied strictly with the research protocol. The study protocol was approved by the Ethics Review Committee of Shanxi Provincial Children's Hospital (IRB-KYYN-202 5-G013), and conformity with the ethical principles specified in the Declaration of Helsinki and its later amendments. The requirement for informed consent was waived because the study involved retrospective review of anonymized clinical data.

### Statistical analysis

Categorical data were evaluated using the chi-square (*χ*^2^) test. Continuous variables underwent parametric analysis (independent-samples *t*-test) when assumptions such as normality and homogeneity of variances were satisfied; otherwise, nonparametric approaches (Mann–Whitney *U* test) were applied. All statistical tests were two-sided, and *P* values <0.05 were considered indicative of statistical significance.

Multivariable logistic regression analysis was performed to examine the association between diverticulum width and gastrointestinal hemorrhage in children presenting with symptomatic Meckel's diverticulum, generating adjusted odds ratios (OR) with their respective 95% confidence intervals (CI). Model 1 was adjusted for sex and age. Model 2 was adjusted for the factors included in Model 1, as well as diverticular height, WBC, albumin and heterotopic mucosa, Mesodiverticular band, Distance from the ileocecal junction. Sensitivity analysis was undertaken to validate and enhance the reliability of the study outcomes. Logistic regression modeling incorporated diverticular width as a discrete variable, alongside the application of a trend test.

To explore the potential nonlinear dose-response effect of Meckel's diverticulum width on gastrointestinal bleeding, a multivariable-adjusted restricted cubic spline model with 4 knots (located at the 5th, 35th, 65th and 95th percentiles) was created to derive the OR curves. A two-piecewise logistic regression model was developed to assess the relationship between Meckel’ diverticular width and gastrointestinal bleeding, adjusting for the potential confounders incorporated into model 2. Additionally, Interaction and subgroup analyses stratified by sex, age (≤3 vs. >3 years) (10), hemoglobin (≤110 vs. >110 g/L), albumin (≤40 vs. >40 g/L), and histopathology (positive vs. negative) were performed to examine the stability of the Meckel's diverticulum width-gastrointestinal bleeding link in different patient groups. Heterogeneity among subgroups and interaction effects were evaluated via logistic regression coupled with likelihood ratio tests (LRT).

The entire statistical analysis was carried out with R software (Version 4.2.0, http://www.R-project.org, The R Foundation) and Free Statistics analysis platform (Version 1.9, Beijing, China, http://www.clinicalscientists.cn/freestatistics). FreeStatistics is a software package that provides intuitive interfaces for most common analyses and data visualization. Most analyses can be done with just a few clicks. It is designed for reproducible analysis and interactive computing (10). *P* < 0.05 (two-tailed) was declared significant.

## Results

### Baseline characteristics

Of the 435 children with MD, 310 children with symptomatic Meckel's diverticulum were eligible for inclusion in the study. The operative approach was open surgery in 157 cases and laparoscopic-assisted surgery in 153 cases. A total of 310 children with symptomatic paediatric Meckel's diverticulum, aged between 0.33 and 15 years, were included in the analysis (Table 1). Of the 310 children with symptomatic paediatric Meckel's diverticulum, 215 were boys and 95 were girls, resulting in a male-to-female ratio of 2.26:1. The overall prevalence of gastrointestinal bleeding disease associated with symptomatic paediatric Meckel's diverticulum was 37.74% (117/310) (Supplementary Table S1). The baseline characteristics of the study cohort, stratified into three equal groups based on Meckel's diverticulum width tertiles, are summarized in Table 1. Significant differences were observed among the three groups in terms of age, bloody stool, Meckel's diverticular height, gastrointestinal bleeding, histopathology, haemoglobin and RBC (*P* < 0.05). Otherwise, the baseline characteristics of the patients were similar (*P* > 0.05).

**Table 1:** Demographic and clinical characteristics of 310 children with symptomatic paediatric Meckel's diverticulum at a single tertiary centre in China.

Variables	Total	Meckel’ diverticular width (cm)			*P*
		T1 (≤0.5 to ≤0.9)	T2 (≤1.0 to ≤1.4)	T3 (≤1.5 to ≤5.0)	
Sex, *n* (%)					0.93
Male	215 (69.4)	32 (71.1)	75 (68.2)	108 (69.7)	
Female	95 (30.6)	13 (28.9)	35 (31.8)	47 (30.3)	
Age (years)	5.0 (2.4, 9.0)	2.9 (1.2, 5.0)	4.0 (1.9, 8.0)	7.0 (3.0, 10.0)	<0.001
Symptom					
Bloody stool, *n* (%)					0.001
No	185 (59.7)	28 (62.2)	51 (46.4)	106 (68.4)	
Yes	125 (40.3)	17 (37.8)	59 (53.6)	49 (31.6)	
Vomiting, *n* (%)					0.481
No	170 (54.8)	25 (55.6)	65 (59.1)	80 (51.6)	
Yes	140 (45.2)	20 (44.4)	45 (40.9)	75 (48.4)	
Abdominal bloating, *n* (%)					0.816
No	285 (91.9)	41 (91.1)	100 (90.9)	144 (92.9)	
Yes	25 (8.1)	4 (8.9)	10 (9.1)	11 (7.1)	
Fever, *n* (%)					0.885
No	247 (79.7)	36 (80)	86 (78.2)	125 (80.6)	
Yes	63 (20.3)	9 (20)	24 (21.8)	30 (19.4)	
Gastrointestinal bleeding, *n* (%)					<0.001
No	193 (62.3)	29 (64.4)	52 (47.3)	112 (72.3)	
Yes	117 (37.7)	16 (35.6)	58 (52.7)	43 (27.7)	
Morphometric parameters of Meckel's diverticulum					
Meckel’ Diverticular height (cm)	3.2 ± 1.5	2.2 ± 1.3	2.9 ± 1.2	3.7 ± 1.5	<0.001
Mesodiverticular band, *n* (%)					0.744
No	228 (73.5)	31 (68.9)	82 (74.5)	115 (74.2)	
Yes	82 (26.5)	14 (31.1)	28 (25.5)	40 (25.8)	
Distance from the ileocecal junction (cm)	54.4 ± 20.4	48.7 ± 18.7	53.8 ± 18.1	56.5 ± 22.2	0.071
Histopathology, *n* (%)					0.0236
Normal ileal mucosa	116 (37.7)	22 (48.9)	38 (34.9)	56 (36.4)	
Heterotopic mucosa	192 (62.3)	23 (51.1)	71 (65.1)	98 (63.6)	
Laboratory examination					
Hemoglobin (g/L)	107.2 ± 32.2	109.6 ± 32.5	100.6 ± 31.7	111.1 ± 31.9	0.027
WBC (×10^9^/L)	10.9 ± 5.1	11.0 ± 5.5	10.7 ± 4.7	10.9 ± 5.3	0.91
Neutrophil percentage (%)	63.6 ± 19.6	60.4 ± 22.6	61.3 ± 19.2	66.3 ± 18.8	0.061
Absolute neutrophil count (×10^9^/L)	6.1 (3.6, 9.5)	5.9 (3.6, 9.7)	5.6 (3.4, 8.6)	6.8 (3.8, 10.3)	0.355
RBC (×10^12^/L)	4.0 ± 1.1	4.1 ± 1.1	3.8 ± 1.1	4.1 ± 1.0	0.027
Platelet (×10^9^/L)	348.7 ± 116.7	341.4 ± 128.4	350.4 ± 121.2	349.5 ± 110.5	0.902
C-reactive protein (mg/L)	2.7 (0.5, 45.4)	2.1 (0.5, 24.2)	1.9 (0.5, 18.5)	3.5 (0.5, 65.8)	0.28
Albumin (g/L)	39.5 ± 5.8	38.0 ± 6.0	39.2 ± 5.6	40.1 ± 5.9	0.081

Data presented are mean ± SD, median (Q1–Q3), or *N* (%); RBC, red blood cell counts; WBC, white blood cell count; T, tertiles.

Within the cohort of 192 pediatric cases showing ectopic mucosa, 176 demonstrated gastric ectopia. Within this group, the number of cases across the tertiles of diverticulum width were 22, 67 and 87, respectively. Additionally, ectopic pancreatic mucosa was present in 16 cases.

### Meckel' diverticular width and gastrointestinal bleeding

Following adjustment for sex, age, diverticular height, white blood cell count, albumin level, histopathology, mesodiverticular band, and distance from the ileocecal junction, a larger diverticular width was inversely associated with gastrointestinal bleeding risk [Adjusted Odds Ratio (aOR) 0.39, 95% CI 0.21–0.72, *P* = 0.002] This inverse association persisted when diverticular width was analyzed by quintiles. Employing the third quintile (Q3) for reference within the adjusted model, the risk of gastrointestinal bleeding is relatively low in the lower quintiles (Q1: aOR 0.28, 95% CI 0.09–0.88, *P* = 0.030; Q2: aOR 0.33, 95% CI 0.11–0.94, *P* = 0.038) and higher width quintiles (Q4: aOR 0.16, 95% CI 0.06–0.46, *P* = 0.001; Q5: aOR 0.06, 95% CI 0.02–0.20, *P* < 0.001) (Table 2).

**Table 2:** Multivariable logistic regression analyses of Meckel’ diverticular width and gastrointestinal bleeding.

Variable	OR (95% CI)					
	Crude	*P* value	Model 1	*P* value	Model 2	*P* value
Meckel’ diverticular width (cm)	0.62 (0.42–0.92)	0.018	0.58 (0.39–0.88)	0.01	0.39 (0.21–0.72)	0.002
Diverticular width quintiles						
Q3 (≤1.2 to ≤1.4)	1 (Ref)		1 (Ref)		1 (Ref)	
Q1 (≤0.2 to ≤0.9)	0.23 (0.1–0.56)	0.001	0.24 (0.1–0.59)	0.002	0.28 (0.09–0.88)	0.030
Q2 (≤1.0 to ≤1.1)	0.29 (0.13–0.65)	0.003	0.3 (0.13–0.68)	0.004	0.33 (0.11–0.94)	0.038
Q4 (≤1.5 to ≤1.8)	0.21 (0.09–0.47)	<0.001	0.2 (0.09–0.45)	<0.001	0.16 (0.06–0.46)	0.001
Q5 (≤2.0 to ≤5.0)	0.12 (0.05–0.28)	<0.001	0.11 (0.05–0.26)	<0.001	0.06 (0.02–0.20)	<0.001
*P* for Trend		0.022		0.012		0.002

OR, odds ratio; CI, confidence interval; Ref, reference; Q, quintiles crude: no other covariates were adjusted. Model 1: we adjusted sex and age. Model 2: we adjusted sex, age, diverticular height, WBC, albumin, histopathology, mesodiverticular band and distance from the ileocecal junction.

### Inverted U-shaped relationship between diverticular width and gastrointestinal bleeding in symptomatic Meckel's diverticulum children

**Figure 2:**
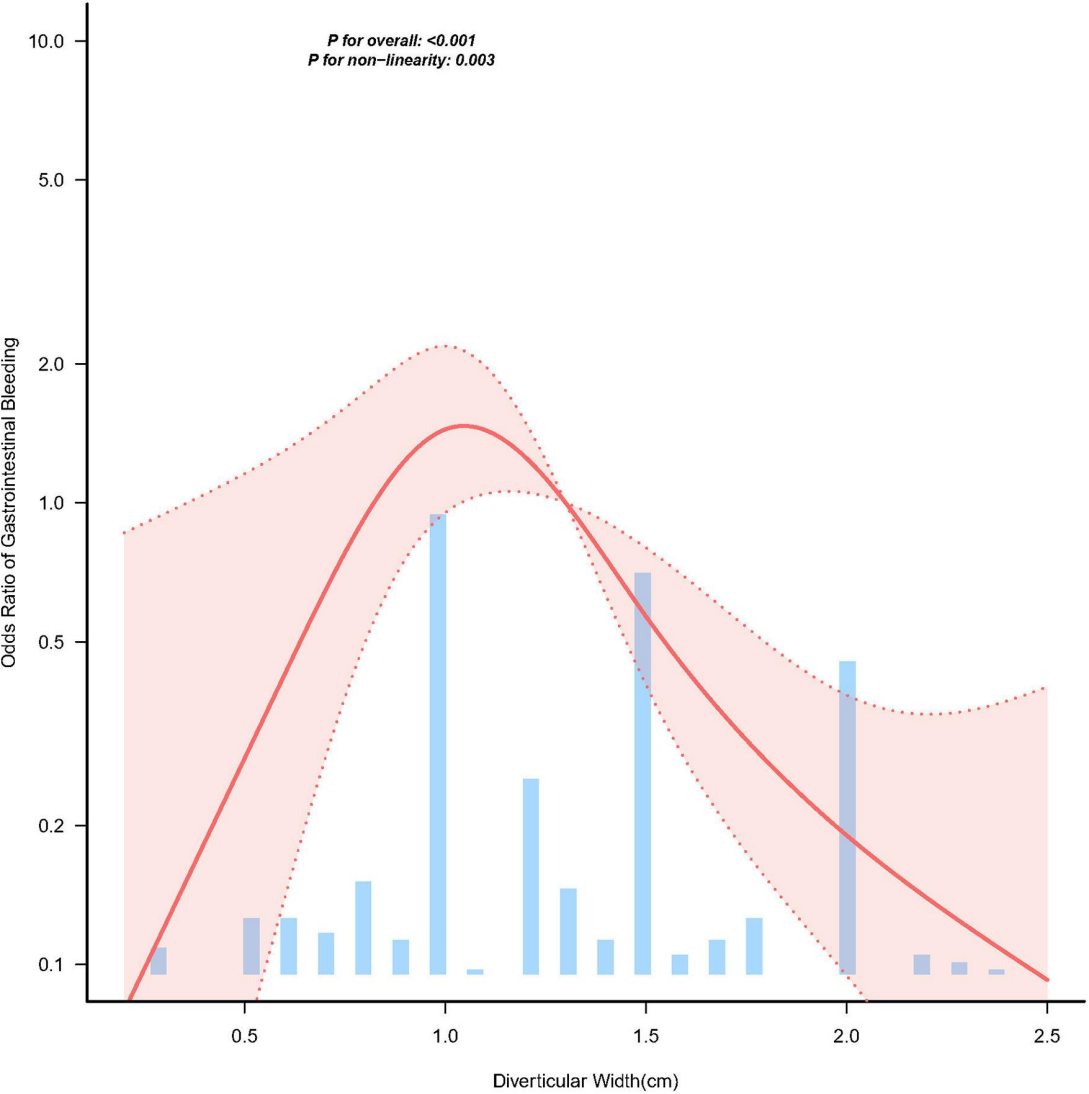
Spline plots for the associations of Meckel' diverticular width and gastrointestinal bleeding. Data were fit by a multivariable logistic regression model based on restricted cubic splines. Meckel' diverticular width was entered as continuous variable. Adjustment factors included sex, age, diverticular height, WBC, albumin, histopathology, mesodiverticular band and distance from the ileocecal junction. Solid and dashed lines represent the predicted value and 95% confidence intervals. Only 95% of the data is shown.

Smooth spline analysis revealed an inverted U-shaped relationship between diverticular width and the risk of gastrointestinal bleeding in paediatric patients with symptomatic Meckel's diverticulum (*P* for non-linearity <0.001; Figure 2). The peak bleeding risk occurred at a diverticular width of 1.25 cm, identified using a two-piecewise linear regression model.

Within the adjusted models, gastrointestinal bleeding hemorrhage risk rose markedly concomitant with increasing diverticulum width at values under the inflection point (OR 12.461, 95% CI 1.333–116.448, *P* = 0.0269). Conversely, for widths above the inflection point, the risk decreased significantly with increasing width (OR 0.287, 95% CI 0.091–0.908, *P* = 0.0336). Findings demonstrate that diminished and elevated diverticular widths correlate with diminished gastrointestinal hemorrhage risk (Supplementary Table S2).

### Subgroup analysis

**Figure 3:**
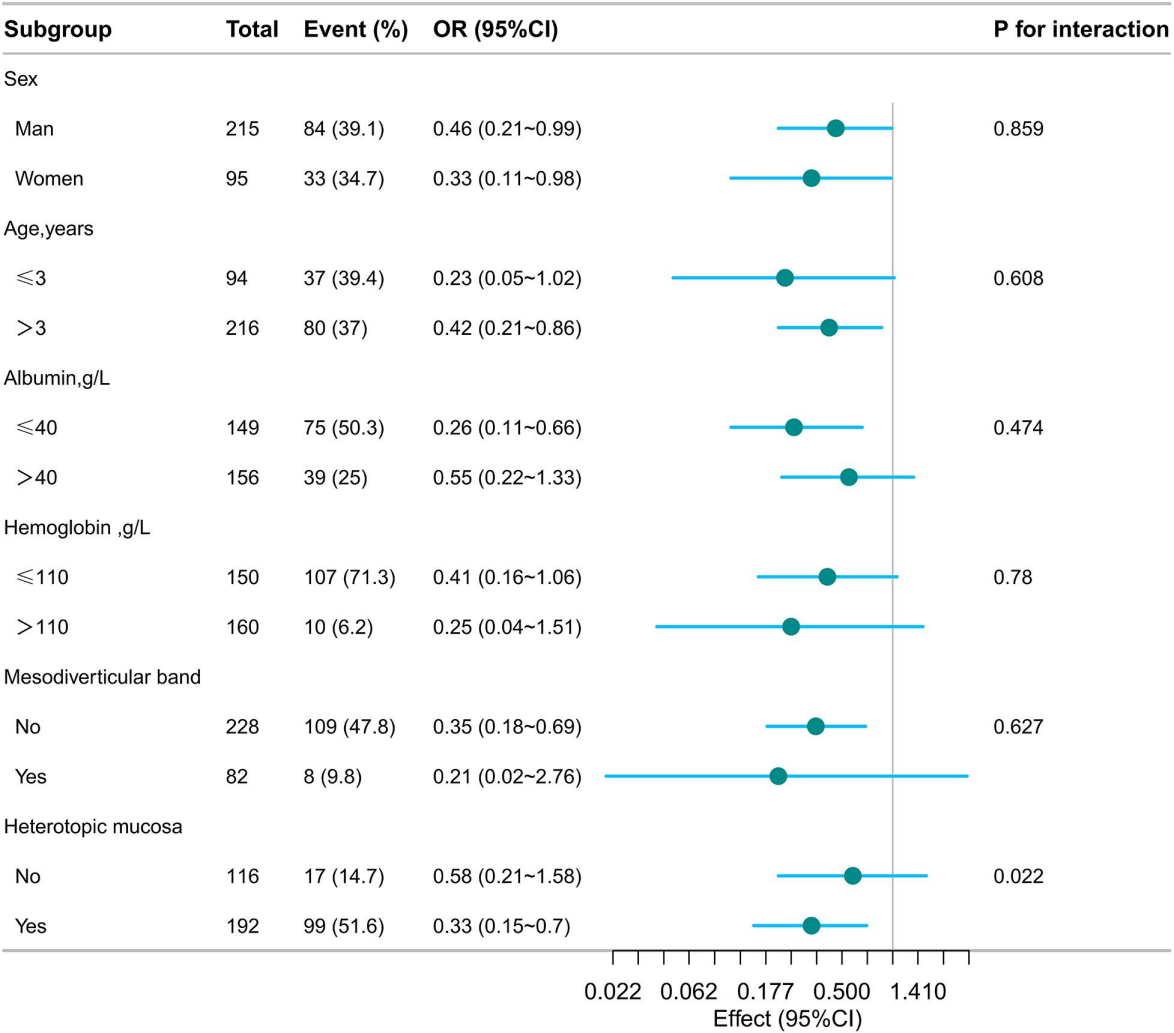
Subgroup analysis of the association between Meckel' diverticular width and gastrointestinal bleeding by baseline characteristics. Except for the stratification factor itself, the stratifications were adjusted for all variables (sex, age, diverticular height, WBC, albumin, histopathology, mesodiverticular band and distance from the ileocecal junction).

To determine the consistency of association between diverticular width and gastrointestinal bleeding across different subgroups of paediatric patients with symptomatic Meckel's diverticulum, we performed stratified and interaction analyses (Figure 3). Grouping patients according to sex, age category (≤3 or >3 years), hemoglobin (≤110 or >110 g/L), albumin (≤39 or >39 g/L), mesodiverticular band status (present/absent), and histopathology, a statistically significant interaction term was identified between histopathology and diverticular width regarding the risk of gastrointestinal bleeding. Among the additional subgroups, interactions were not significant.

## Discussion

This study provides novel insights into the relationship between Meckel's diverticulum morphology and gastrointestinal bleeding risk in children. We establish for the first time that diverticular width independently predicts gastrointestinal bleeding risk through an inverted U-shaped association, with peak risk occurring at 1.25 cm. Furthermore, we identify a significant interaction between ectopic mucosa and diverticular width in modulating bleeding risk. These findings demonstrate significant clinical relevance for pediatric surgeons managing symptomatic Meckel's diverticulum.

Our demonstration of an independent association between diverticular width and gastrointestinal bleeding risk persists after rigorous adjustment for established confounders, including histopathology, diverticular length, and mesodiverticular bands. The literature has largely emphasized Meckel diverticulum length (5) or its height-to-diameter ratio (6, 11–13). To date, no studies have investigated the link between Meckel's diverticulum width and gastrointestinal bleeding. In a study of 88 paediatric patients, Slívová et al. (14) demonstrated that those with ectopic gastric tissue exhibited significantly wider diverticulum bases (2.1 ± 0.57 vs. 1.2 ± 0.41 cm, *P* < 0.001). Based on their analysis, the authors determined that the base width of Meckel's diverticulum serves as a significant indicator for the presence of heterotopic gastric mucosa. Furthermore, diverticula measuring ≥1.5 cm in diameter demonstrated a substantially elevated likelihood of containing such ectopic tissue. In contrast to Slívová et al.'s findings, however, a retrospective analysis of 111 paediatric MD cases in China revealed no significant difference in diverticulum base width between the ectopic mucosa group and the non-ectopic group (1.5 ± 0.6 vs. 1.4 ± 0.7 cm, *P* = 0.39) (2). These research discrepancies may be related to ethnicity. Multiple investigations have established a strong association between heterotopic gastric mucosa and symptomatic presentations of Meckel's diverticulum, especially gastrointestinal hemorrhage (1, 2, 11, 15). Nonetheless, physician reliance on direct visual or tactile examination alone proves inadequate for identifying ectopic mucosa within the diverticulum. Consequently, identifying predictive clinical markers is necessary. This research uncovers a novel, non-monotonic (inverted U-shaped) association linking diverticulum width to gastrointestinal bleeding risk, and demonstrates that there is a significant interaction between ectopic tissue and diverticular width that regulates the risk of digestive tract bleeding.

Based on previous studies and our own research, we have proposed a potential mechanism that links the width of Meckel's diverticulum to gastrointestinal bleeding. A diverticulum width measuring less than 1.25 cm is associated with an elvated risk of gastrointestinal bleeding that intensifies proportionally as the dimension increases. This may be due to insufficient lumen volume and minimal ectopic gastric mucosa preventing significant accumulation of gastric acid and ulcer formation, which is insufficient to trigger substantial bleeding. Conversely, when the width of the diverticulum exceeds 1.25 cm, the risk of gastrointestinal bleeding decreases as it widens further. This may be because diverticulum contents, such as gastric acid secreted by ectopic gastric mucosa, can be effectively expelled, thereby minimising retention. This reduces the risk of irritation to the diverticulum neck or its own mucosa, thereby decreasing ulcer formation and lowering the risk of bleeding. Children exhibiting larger Meckel diverticulum dimensions demonstrate a greater prevalence of ectopic gastric mucosa. Heterotopic mucosa, particularly gastric type, represents a primary pathogenic mechanism underlying gastrointestinal hemorrhage (2, 16). Meckel's diverticulum causing clinical symptoms exhibits a strong male predominance (15–17). This gender disparity is attributable to elevated gastrin and gastric acid concentrations in males, which predispose to symptom manifestation (15, 18). Such evidence further substantiates the contribution of ectopic gastric tissue to the development of clinical manifestations in children with Meckel's diverticulum. Our cohort revealed a male-to-female ratio of 2.26 for symptomatic cases. Gastrointestinal bleeding accounted for 37.74% of cases (117/310). In children presenting with gastrointestinal hemorrhage, histologically confirmed heterotopic mucosa was identified in 84.62% of cases, aligning with findings documented in prior published reports. This study demonstrates an inverted U-shaped relationship between diverticulum diameter and the risk of gastrointestinal bleeding. This provides morphological evidence for assessing bleeding risk in cases of Meckel's diverticulum. Specifically, the presence of ectopic mucosa appears to increase susceptibility to gastrointestinal bleeding within a certain range of diverticulum width.

Acknowledgment of certain limitations is warranted for this study. Chief among them is the potential for selection bias inherent in its retrospective nature and confinement to a single institution; however, our strict inclusion criteria mitigated this concern. Secondly, there may be inter-observer variability when measuring diverticulum width. Thirdly, the lack of quantitative data on mucosal surface area and acid secretion means that more robust evidence of the underlying mechanisms cannot be provided. Finally, although Meckel's diverticulum dimensions are congenital, the lack of standardized, validated dietary data in our retrospective cohort is a limitation. To improve clinical interpretability, large-scale multicentre standardised studies are urgently needed for validation.

## Conclusions

We have established that diverticular width is an independent predictor of gastrointestinal bleeding risk in paediatric Meckel's diverticulum, characterised by an inverted U-shaped relationship with a peak risk at 1.25 cm. The significant interaction between ectopic mucosa and diverticular width reveals distinct pathophysiological pathways. Utilization of these anatomical parameters improves risk categorization in children and underpins personalized, evidence-driven surgical management strategies.

## Data Availability

The datasets presented in this article are not readily available because data supporting the findings of this study are available upon reasonable request to the corresponding author. Requests to access the datasets should be directed to Hongwei Xi, xihongwei148@sina.com.
