# Supplementary material for: Inverted U-shaped relationship between diverticular width and gastrointestinal bleeding in symptomatic Meckel's diverticulum children: a retrospective single-center study
Source: Front Pediatr. 2026 May 15;14:1774572. doi: 10.3389/fped.2026.1774572 (PMC13219009; doi:10.3389/fped.2026.1774572)
Supplement: Supplementary file 1 [file Table1.docx]

| **Variables** | **Total**  **(n = 310)** | **Non-Gastrointestinal bleeding**  **(n = 193)** | **Gastrointestinal bleeding**  **(n = 117)** | ***P*** |
| --- | --- | --- | --- | --- |
| Sex, n (%) |  |  |  | 0.468 |
| Male | 215 (69.4) | 131 (67.9) | 84 (71.8) |  |
| Female | 95 (30.6) | 62 (32.1) | 33 (28.2) |  |
| Age(years) | 5.0 (2.4, 9.0) | 5.0 (2.6, 9.0) | 5.0 (2.0, 9.0) | 0.954 |
| **Symptom** |  |  |  |  |
| Bloody stool, n (%) |  |  |  | < 0.001 |
| No | 185 (59.7) | 185 (95.9) | 0 (0) |  |
| Yes | 125 (40.3) | 8 (4.1) | 117 (100) |  |
| Vomiting, n (%) |  |  |  | < 0.001 |
| No | 170 (54.8) | 64 (33.2) | 106 (90.6) |  |
| Yes | 140 (45.2) | 129 (66.8) | 11 (9.4) |  |
| abdominal bloating, n (%) |  |  |  | < 0.001 |
| No | 285 (91.9) | 168 (87) | 117 (100) |  |
| Yes | 25 ( 8.1) | 25 (13) | 0 (0) |  |
| Fever, n (%) |  |  |  | < 0.001 |
| No | 247 (79.7) | 139 (72) | 108 (92.3) |  |
| Yes | 63 (20.3) | 54 (28) | 9 (7.7) |  |
| **Morphometric parameters of Meckel's diverticulum** | | |  |  |
| Diverticular height(cm) | 3.2 ± 1.5 | 3.3 ± 1.5 | 2.9 ± 1.4 | 0.027 |
| Diverticular width（cm） | 1.5 ± 0.6 | 1.5 ± 0.7 | 1.3 ± 0.5 | 0.016 |
| mesodiverticular band, n (%) |  |  |  | < 0.001 |
| No | 228 (73.5) | 119 (61.7) | 109 (93.2) |  |
| Yes | 82 (26.5) | 74 (38.3) | 8 (6.8) |  |
| Distance from the ileocecal junction（cm） | 54.4 ± 20.4 | 52.2 ± 20.5 | 58.1 ± 19.9 | 0.013 |
| **Histopathology, n (%)** |  |  |  | < 0.001 |
| Normal ileal mucosao | 116 (37.7) | 99 (51.6) | 17 (14.7) |  |
| Heterotopic mucosa | 192 (62.3) | 93 (48.4) | 99 (85.3) |  |
| **Laboratory examination** |  |  |  |  |
| Hemoglobin (g/L) | 107.2 ± 32.2 | 124.0 ± 24.6 | 79.4 ± 22.7 | < 0.001 |
| WBC (×10^9^/L) | 10.9 ± 5.1 | 11.9 ± 5.4 | 9.1 ± 4.0 | < 0.001 |
| Neutrophil percentage(%) | 63.6 ± 19.6 | 70.7 ± 17.8 | 52.1 ± 17.0 | < 0.001 |
| Absolute neutrophil count (×10^9^/L) | 6.1 (3.6, 9.5) | 7.7 (4.8, 12.5) | 3.9 (2.8, 6.1) | < 0.001 |
| RBC(×10^12^/L) | 4.0 ± 1.1 | 4.6 ± 0.7 | 3.1 ± 0.8 | < 0.001 |
| Platelet (×10^9^/L) | 348.7 ± 116.7 | 358.7 ± 115.2 | 332.1 ± 117.8 | 0.052 |
| C-reactive protein(mg/L) | 2.7 (0.5, 45.4) | 12.3 (1.1, 74.3) | 0.5 (0.5, 1.5) | < 0.001 |
| Albumin(g/L) | 39.5 ± 5.8 | 40.4 ± 6.0 | 37.9 ± 5.2 | < 0.001 |

Table S1: Comparison of general and biochemical characteristics between gastrointestinal bleeding and non-gastrointestinal bleeding groups.

Data presented are mean ± SD, median (Q1-Q3), or N (%);RBC, red blood cell counts; WBC, white blood cell count;

Table S2.Threshold effect analysis of Meckel' diverticular width on gastrointestinal bleeding

| **Meckel' Diverticular width** | **OR(95%CI)** | ***P* value** |
| --- | --- | --- |
| **＜1.25cm** | 12.461 (1.333~116.448) | 0.0269 |
| **≥1.25cm** | 0.195 (0.047~0.803) | 0.0236 |
| **Likelihood Ratio test** |  | <0.001 |

Adjustment factors included sex, age, diverticular height, WBC, albumin, Histopathology, Mesodiverticular band and Distance from the ileocecal junction Only 95% of the data is shown.
